# Health-resource use and quality of life in children with bronchiectasis: a multi-center pilot cohort study

**DOI:** 10.1186/s12913-019-4414-5

**Published:** 2019-08-13

**Authors:** Yolanda G. Lovie-Toon, Keith Grimwood, Catherine A. Byrnes, Vikas Goyal, Greta Busch, I. Brent Masters, Julie M. Marchant, Helen Buntain, Kerry-Ann F. O’Grady, Anne B. Chang

**Affiliations:** 1 Institute of Health & Biomedical Innovation @ Centre for Children’s Health Research, Queensland University of Technology, 62 Graham Street, South Brisbane, Queensland 4101 Australia; 2 School of Medicine and Menzies Health Institute Queensland, Griffith University, Gold Coast, Queensland 4222 Australia; 3 Departments of Infectious Diseases and Paediatrics, Gold Coast Health, Gold Coast, Queensland 4215 Australia; 4 Department of Paediatrics, University of Auckland, Auckland, New Zealand; 5 Respiratory Department, Starship Children’s Hospital, Auckland, New Zealand; 6 Department of Respiratory Medicine, Queensland Children’s Hospital, South Brisbane, Queensland 4101 Australia; 7 School of Medicine, The University of Queensland, Herston, Queensland 4006 Australia; 8 Child Health Division, Menzies School of Health Research, Tiwi, Northern Territory 0810 Australia

**Keywords:** Bronchiectasis, Children, Health-related quality of life, Health resource use

## Abstract

**Background:**

Bronchiectasis in children is an important, but under-researched, chronic pulmonary disorder that has negative impacts on health-related quality of life. Despite this, it does not receive the same attention as other chronic pulmonary conditions in children such as cystic fibrosis. We measured health resource use and health-related quality of life over a 12-month period in children with bronchiectasis.

**Methods:**

We undertook a prospective cohort study of 85 children aged < 18-years with high-resolution chest computed-tomography confirmed bronchiectasis undergoing management in three pediatric respiratory medical clinics in Darwin and Brisbane, Australia and Auckland, New Zealand. Children with cystic fibrosis or receiving cancer treatment were excluded. Data collected included the frequency of healthcare attendances (general practice, specialists, hospital and/or emergency departments, and other), medication use, work and school/childcare absences for parents/carers and children respectively, and both parent/carer and child reported quality of life and cough severity.

**Results:**

Overall, 951 child-months of observation were completed for 85 children (median age 8.7-years, interquartile range 5.4–11.3). The mean (standard deviation) number of exacerbations was 3.3 (2.2) per child-year. Thirty of 264 (11.4%) exacerbation episodes required hospitalization. Healthcare attendance and antibiotic use rates were high (30 and 50 per 100 child-months of observation respectively). A carer took leave from work for 53/236 (22.5%) routine clinic visits. Absences from school/childcare due to bronchiectasis were 24.9 children per 100 child-months. Quality of life scores for both the parent/carer and child were highly-correlated with one another, remained stable over time and were negatively associated with cough severity.

**Conclusions:**

Health resource use in this cohort of children is high, reflecting their severe disease burden. Studies are now needed to quantify the direct and societal costs of disease and to evaluate interventions that may reduce disease burden, particularly hospitalizations.

**Electronic supplementary material:**

The online version of this article (10.1186/s12913-019-4414-5) contains supplementary material, which is available to authorized users.

## Background

Bronchiectasis is a chronic pulmonary disorder characterized by persistent wet or productive cough, recurrent exacerbations and abnormal dilatation of bronchial airways [1]. The prevalence in children is highest in low-income countries and Indigenous populations [2, 3], and the adverse impact on the child and carer’s quality of life (QoL) is increasingly recognised [4].

Illnesses that consume extensive health resources attract the attention of policy makers and funders [5]. Consequently, the European Multicentre Bronchiectasis Audit and Research Collaboration has recently identified healthcare utilisation as a research priority [6]. However, in children with bronchiectasis few studies describe its impact upon individuals and healthcare services. The limited published data from Australia and New Zealand are derived from hospital cohort studies [7, 8] or clinical trials [9], which cannot be generalized to out-patient or community settings. While in New Zealand the estimated cost of pediatric bronchiectasis hospitalizations was NZD 0.95 million in the 2012/13 financial year [8], there are no similar published Australian data on healthcare service utilization or costs related to bronchiectasis.

Thus, with the aim of informing government healthcare services and future research, our primary objective was to estimate healthcare resource use over a 12-month period for children aged < 18-years with bronchiectasis receiving ongoing care in pediatric respiratory medical centers. Our secondary objective was to measure and correlate the health-related QoL of parents/carers and children with bronchiectasis.

## Methods

### Study design

Eligible children with bronchiectasis were recruited from respiratory clinics in two Australian (Brisbane and Darwin) and one New Zealand (Auckland) public hospitals between December 2012 and January 2016 and followed for 12-months. Inclusion criteria were aged < 18-years, chest computed-tomography confirmed bronchiectasis and cared for by a respiratory physician. Children with cystic fibrosis, enrolled in another study or receiving treatment for cancer were excluded. Research nurses approached potential participants during routine respiratory clinic appointments to obtain consent to participate.

### Data collection

Demographic and bronchiectasis-related characteristics were collected upon enrolment. Research nurses conducted monthly reviews by phone, email or in person, where they recorded types of healthcare sought and school/childcare/work absences due to bronchiectasis, as well as any medications taken and exacerbations in the previous month. An exacerbation was defined as the child being unwell for ≥3-days with at least one of the following symptoms: an increased cough, change in cough quality, or increased sputum volume or purulence [10]. This definition has been used in randomized controlled trials in pediatric bronchiectasis [9, 11]. All exacerbations were reported by parent/carers who had been instructed by research staff on the symptoms constituting the study definition of an exacerbation. When an exacerbation was reported, additional information on resource use for the exacerbation was collected from the parent/carer. All resource utilisation collected through monthly reviews were parent-reported and not confirmed by other sources (e.g. medical records). Face-to-face-clinical visits were scheduled quarterly in accordance with routine clinical practice at the participating hospitals.

Parent/carer-reported and/or child-reported QoL and cough severity were assessed using validated tools at baseline and at months 3, 6, 9 and 12. These data were collected either face-to-face at clinic visits, or through telephone interviews. Parent/carers completed the Parent Cough-specific QoL questionnaire (PC-QoL) [12] and children aged > 7-years completed the Child Cough-specific QoL questionnaire (CC-QoL) [13]. In both tools, the QoL scores range from 1 (low QoL) to 7 (high QoL). Cough severity was measured using a cough score (0 = no cough to 5 = severe cough and cannot perform usual activities) [14].

### Sample size

As this was a pilot study, a specific sample size was not calculated. The target enrolment was 100 children, to achieve a complete dataset on 80 children.

### Data analysis

Baseline demographic and bronchiectasis-related characteristics were analyzed descriptively and expressed as counts and proportions, means (standard deviation [SD]) or medians (inter-quartile range [IQR]). Resource use was summarized as the number and proportion of children reporting each type of resource use in the past month, during exacerbations, and at routine clinic visits. Rates of resource use over the 12-months were calculated as the number of children using a particular resource per 100 child-months of observation. Exacerbation-related resource use was summarized for all exacerbations and by season of exacerbation onset. Season of exacerbation onset was categorized as spring (September–November), summer (December–February), autumn (March–May), and winter (June–August) using date of exacerbation onset. Parent/carer and child-reported QoL was summarized by each clinical visit. The Pearson or Spearman correlation coefficients were used to analyze relationships between parent- and child-reported QoL, parent- and child-reported cough severity, and parent- and child-reported QoL against parent- and child-reported cough severity.

## Results

### Participant baseline characteristics

The target of 100 enrolled children was reached. The data for 15 were considered too unreliable for meaningful analysis, due to reporting errors or situations where study procedures had not been followed. These participants were subsequently excluded.

The 85 children (42 female, 34 Indigenous Australian/New Zealand subjects) were recruited from the Queensland Children’s Hospital (*n* = 47), Auckland Starship Children’s Hospital (*n* = 26) and Royal Darwin Hospital (*n* = 12). The median age at enrolment was 8.7 (IQR 5.4–11.3) years and their median age at diagnosis of bronchiectasis was 3.6 (IQR 2.1–6.5) years. The median time since diagnosis was 2.9 (IQR 1.7–6.1) years with a mean of 2.6 (SD 1.1) lobes affected. The primary etiology was post-infectious (*n* = 51), followed by idiopathic (*n* = 14), aspiration (*n* = 7) and other (*n* = 13).

### Monthly reviews

Overall, 951 monthly follow-up reviews were completed over the study period. Completions declined over the 12-months (Table 1). There was a greater proportion of children aged  < 5 years, with a low birthweight (< 2500 g) and who were born pre-term (gestational age < 37-weeks) among participants who completed all 12-monthly follow-ups, compared to participants who did not (Additional file 1: Table S3). On average, within the cohort 30 children/100-months of observation attended a general practitioner (GP), emergency department (ED) or pediatric specialist physician for their bronchiectasis, while the corresponding rate for other health reasons was 17 children/100-months. The number of attendances per child per month was not collected. More children presented to a specialist for their management than GPs, and EDs were used infrequently. In any given month, over half of the children were taking some form of medication. Medication use was not specified for bronchiectasis only, and the number of courses used per month was not collected.

**Table 1:** Monthly medical resource use, missed school and childcare, and missed parent/carer work (*N* = 85 children)

	Month 1	Month 2	Month 3	Month 4	Month 5	Month 6	Month 7	Month 8	Month 9	Month 10	Month 11	Month 12	TOTAL	Per 100 child-mths
Number of follow-ups completed	82	84	82	83	80	80	79	78	78	78	75	72	951	N/A
Saw GP														
For bronchiectasis	7	7	8	11	10	14	11	13	9	12	8	8	118	12.4
For other reason	4	8	6	7	5	3	6	8	6	2	3	7	65	6.8
Saw ED														
For bronchiectasis	5	3	2	1	1	0	2	1	2	0	1	0	17	1.8
For other reason	3	0	0	1	0	2	1	0	0	0	1	1	9	1.0
Saw specialist														
For bronchiectasis	11	7	12	14	15	14	9	12	12	14	13	14	147	15.5
For other reason	6	10	9	7	5	10	8	8	7	6	7	6	89	9.4
Had medications *for any reason*	51	47	48	53	51	52	48	52	46	48	43	40	579	60.9
Had antibiotics *for any reason*	37	34	35	40	37	39	36	42	36	37	35	29	437	46.0
Had inhaled corticosteroids *for any reason*	9	13	11	15	11	12	12)	11	12	9	6	8	129	13.6
Missed school/ childcare	21	17	15	20	22	26	18	22	19	21	20	16	237	24.9
Primary carer missed work	10	9	9	6	5	10	8	10	13	9	11	10	110	11.6^a^
Secondary carer missed work	4	4	5	2	2	4	1	2	3	3	2	1	33	3.5^a^
Median days missed school/ childcare (IQR)	3 (2–7)	3 (2–5)	4 (2–7)	3 (2–5.5)	3 (2–5)	4 (2.5–7)	3 (1–5)	2.5 (1–4)	4 (1–5)	3 (2–5.5)	3 (1–5)	3.5 (1.5–5.5)	948	12.0/child-year
Median days primary carer missed work (IQR)	2.5 (1.5–4)	2 (1–13)	3 (1.5–7.5)	2 (1.25–2)	2 (2–3)	3 (1.5–4)	1 (1–4)	1.5 (1–2.5)	2 (1–3)	3 (1–4)	2 (1–2.5)	2.5 (1.5–3)	278	3.5 /child-year^a^
Median days secondary carer missed work (IQR)	1 (1–1)	3 (3–3)	1 (1–1)	1 (0–2)	1 (1–1)	1 (0–1)	1 (1–1)	1.5 (1–2)	4 (1–7)	2 (1–2)	1 (1–1)	2 (2–2)	37	0.5/child-year^a^

^a^Only applies to parents/guardians who were in paid employment

*Abbreviations ED* Emergency Department, *GP* general practitioner, *IQR* interquartile range, *N/A* not applicable

In any given month, approximately one-quarter of children within the cohort missed some period of school or childcare due to their bronchiectasis; fewer parents/carers missed work. The median number of days missed per child-month ranged from 2.5 (IQR 1.0–4.0) to 4.0 (IQR 2.0–7.0).

### Exacerbations

Overall, 276 bronchiectasis exacerbations were reported (mean 3.3 [SD 2.2] per child-year). Twelve episodes had missing data for resource use associated with the exacerbation, leaving 264 for analysis (Table 2). Of these, 59 (22%) began in spring, 54 (20%) in summer, 62 (23%) in autumn, and 82 (31%) began in winter, while for 7 (3%) onset season was unknown. Overall when looking solely at exacerbations, GPs were the most frequently used healthcare resource. Thirty episodes resulted in hospitalization; 20/264 (7.6%) were treated exclusively in hospital (mean stay 6.7 [SD 4.8] days), while 10/264 (3.8%) had “hospital-in-the-home” following their inpatient stay (mean length of care 9.0 [SD 2.4] days). Parents reported they had antibiotics at home (or a prescription to fill) in approximately one-third of exacerbations. A bronchoscopy was performed on one occasion. The frequency of resource use varied by season, however variations were inconsistent between different types of healthcare resource use. Frequency of use was highest during spring for GPs and specialists, highest during summer for hospitalizations, and highest during winter for antibiotic consumption.

**Table 2:** Exacerbation-related resource use, total and by season^a^

	All, *n* = 264	Spring, *n* = 59	Summer, *n* = 54	Autumn, *n* = 62	Winter, *n* = 82
	*n (%)*	*n (%)*	*n (%)*	*n (%)*	*n (%)*
General practitioner visit	123 (46.6)	30 (50.9)	26 (48.2)	28 (45.2)	35 (42.7)
Had a prescription at home to fill/had antibiotics at home	77 (29.2)	15 (25.4)	16 (29.6)	16 (25.8)	28 (34.2)
Specialist visit	69 (26.1)	20 (33.9)	12 (22.2)	15 (24.2)	22 (26.8)
Physiotherapy	41 (15.5)	7 (11.9)	9 (16.7)	10 (16.1)	13 (15.9)
Telephone advice from respiratory clinic nurse	32 (12.1)	4 (6.8)	3 (5.6)	12 (19.4)	12 (14.6)
Pathology	30 (11.4)	4 (6.8)	9 (16.7)	9 (14.5)	8 (9.8)
Haematology (full blood count)	15	4	4	5	2
Biochemistry	10	4	3	3	0
Sputum culture	9	1	3	4	1
Antibiotic blood levels	4	0	3	1	0
Throat swab for virus nucleic acid amplification testing	3	0	1	2	0
Nasopharyngeal aspirate for virus nucleic acid amplification testing	1	0	0	0	1
Bronchoalveolar lavage	1	0	0	0	1
Immunology (radioallergoabsorbent test)	1	0	0	1	0
Missing	4	0	0	0	4
In-patient hospital admission	30 (11.4)	4 (6.8)	8 (14.8)	9 (14.5)	8 (9.8)
Had ‘hospital in the home’ following inpatient admission	10	0	4	3	2
Emergency department presentation	25 (9.5)	3 (5.1)	5 (9.3)	6 (9.7)	9 (11.0)
Medical imaging	21 (8.0)	2 (3.4)	5 (9.3)	5 (8.1)	9 (11.0)
Chest radiograph	14	1	4	3	6
Chest computed tomography scan	2	0	1	0	1
Other	7	1	1	2	3
Missing	2	0	0	0	1
Respiratory function testing (spirometry)	16 (6.1)	4 (6.8)	3 (5.6)	4 (6.5)	5 (6.1)
Saw other services (during hospitalisation)	12 (4.6)	3 (5.1)	1 (1.9)	3 (4.8)	5 (6.1)
Social worker	4	1	1	2	0
Dietician	3	1	0	1	1
Occupational therapist	1	0	0	0	1
Cardiologist	1	0	0	0	1
Surgical	1	1	0	0	0
Consult liaison	1	1	0	0	0
Dentist	1	0	0	0	1
Missing	1	0	0	0	1
Over the counter treatments	8 (2.9)	1 (1.7)	2 (3.7)	4 (6.5)	1 (1.2)
Consultation with community health nurse / respiratory clinic nurse	7 (2.7)	1 (1.7)	1 (1.9)	3 (3.2)	3 (3.7)
Hospital school (during hospitalisation)	7 (2.7)	2 (3.4)	0 (0.0)	3 (4.8)	2 (2.4)
Other procedures	2 (0.8)	0 (0.0)	1 (1.9)	0 (0.0)	1 (1.2)
Bronchoscopy	1	0	1	0	0
Scheduled intravenous line maintenance	1	0	0	0	3
Telephone advice from other doctor/specialist	2 (0.8)	1 (1.7)	0 (0.0)	0 (0.0)	1 (1.2)
Alternative therapies	2 (0.8)	1 (1.7)	0 (0.0)	0 (0.0)	1 (1.2)
Glutathione nebulised medication	1	1	0	0	0
Acupuncture	1	0	0	0	1
Chinese medicine	1	0	0	0	1
Telephone advice from general nurse	1 (0.4)	0 (0.0)	0 (0.0)	1 (1.6)	0 (0.0)

^a^There were 7 exacerbations with unknown start dates, and therefore onset season could not be determined

### Clinic visits

Most children (77/85) completed a clinic visit at baseline. Approximately half of the cohort completed clinic visits and the corresponding questionnaire at the four subsequent time-points (Table 3). Consultant respiratory physicians were the most common clinicians present at the review (192/236; 81.4%), while respiratory function testing (154/236; 65.3%) and physiotherapy (85/236; 36.0%) were the most frequently used additional clinical services. Parents/carers took leave from work to attend the appointment in 53/236 (22.5%) clinic visits.

**Table 3:** Resource use at quarterly clinic visits

	Baseline *N* = 77	Month 3 *N* = 40	Month 6 *N* = 48	Month 9 *N* = 36	Month 12 *N* = 35	Total *N* = 236
Visit details						
Consultant Respiratory Physician	58	31	40	30	33	192 (81.4)
Respiratory Fellow (advanced trainee)	17	7	5	3	1	33 (14.0)
Nurse	2	2	3	3	1	11 (4.7)
Additional clinical services utilised						
Respiratory Function testing	51	25	26	26	26	154 (65.3)
Physiotherapy	27	17	16	14	11	85 (36.0)
Other allied health	7	5	4	3	0	19 (8.1)
Respiratory nurse consultant	22	14	12	7	7	62 (26.3)
Pharmacy - Prescription filled	23	14	12	11	7	67 (28.4)
Pathology	11	7	8	4	5	35 (14.8)
Medical Imaging	3	2	1	3	2	11 (4.7)
Box study or exercise challenge	0	1	0	2	1	4 (1.7)
Other consults	3	7	3	3	2	18 (7.6)
Healthcare card^a^						
Yes	60	30	37	25	29	181 (76.7)
No	15	9	10	9	6	49 (20.8)
Missing	2	1	1	2	0	6 (2.5)
Private health insurance						
Yes	23	13	15	15	9	75 (31.8)
No	50	26	31	18	26	151 (64.0)
Missing	4	1	2	3	0	10 (4.2)
Primary carer required to take leave to attend clinic appointment						
Sick/care’s leave taken	10	2	5	6	3	26 (11.0)
No leave required	27	14	21	11	15	88 (37.3)
Unpaid leave taken	7	0	2	4	2	15 (6.4)
Not in paid employment	25	11	12	5	8	61 (25.9)
Did not attend	3	0	1	3	2	9 (3.8)
Other	4	4	3	3	3	17 (7.2)
Missing	1	9	4	4	2	20 (8.5)
Secondary carer required to take leave to attend clinic appointment						
Sick/care’s leave taken	3	1	2	2	2	10 (4.2)
No leave required	7	2	1	3	2	15 (6.4)
Unpaid leave taken	1	1	0	0	0	2 (0.9)
Not in paid employment	1	1	0	2	0	4 (1.7)
Did not attend	41	15	16	13	16	101 (42.8)
Other	1	1	0	1	0	3 (1.3)
Missing/NA	23	19	29	15	15	101 (42.8)
Additional carer required to take leave to attend clinic appointment						
No leave required	1	0	0	0	0	1 (0.4)
Not in paid employment	1	0	0	0	0	1 (0.4)
Did not attend	5	2	3	0	0	10 (4.2)
Missing/NA	70	38	45	36	35	224 (94.9)

^a^A healthcare card is a government-issued card, which provides the cardholder with access to additional subsidies for medicines and/or healthcare services within the public healthcare system. Healthcare cards are issued to individuals who meet certain social and/or economic criteria

### Quality of life and cough severity

Similar to clinic visits, QoL and cough severity questionnaire completions were high at baseline, but decreased at subsequent time-points (Table 4). Whether the child was experiencing an exacerbation at the time the QoL and cough severity questionnaires were completed was not recorded. Median parent/carer and child QoL scores were consistent across all time-points (Table 4). Parent/carer scores ranged from 5.9 (IQR 4.3–6.8) to 6.5 (IQR 5.4–6.9) and child scores ranged from 6.1 (IQR 4.5–6.7) to 6.8 (IQR 5.0–7.0). Median parent/carer and child-reported cough severity scores ranged from 1.0 (IQR 0.0–2.0) to 2.0 (IQR 0.0–2.0) and from 1.0 (IQR 0.0–2.0) to 2.0 (IQR 0.5–2.5), respectively (Additional file 1: Table S1).

**Table 4:** Parent and child-reported cough quality-of-life (QoL) at time of clinic visits

	Parent Cough-QoL (N = 85)		Child Cough-QoL		
	Number completed	Median (IQR)	Number of eligible children (aged > 7-years)	Number completed	Median (IQR)
Baseline	72	6.0 (4.7–6.9)	49	35	6.5 (4.9–7.0)
Month 3	41	6.5 (5.4–6.9)	51	20	6.1 (4.5–6.7)
Month 6	55	5.9 (4.3–6.8)	54	25	6.4 (4.3–7.0)
Month 9	46	5.9 (4.9–7.0)	57	19	6.6 (3.3–6.9)
Month 12	47	5.9 (4.7–7.0)	57	26	6.8 (5.0–7.0)

*Abbreviations*: *IQR* interquartile range

Whilst small numbers of participants completed both PC-QoL and CC-QoL questionnaires at each time-point (Additional file 1: Table S2), there were high and significant positive correlations between parent/carer and child QoL scores at each time-point (*p*-values < 0.002). There were also high positive correlations between parent-reported and child-reported cough severity scores. High negative correlations were observed between child-reported cough severity and QoL scores at each time-point and between parent/carer-reported cough severity and QoL scores.

## Discussion

Here, we described the annual health resource use and parent/carer and child QoL scores in children with bronchiectasis managed at two Australian and one New Zealand hospitals. In this cohort, children averaged three exacerbations per annum. In any given month, approximately one-third of the cohort presented for medical care, half used antibiotics and one-quarter had absences from school or childcare. Parents/carers sought medical care from a specialist more frequently than GPs or EDs, except during exacerbations. While exacerbations were more frequent in winter, seasonal frequency of exacerbation-related resource use varied between types of resource use. Eleven-percent of exacerbations resulted in hospitalization, and in one-third this included subsequent “hospital-in-the-home” care. Although over time QoL scores for both parents/carers and children were high and stable, better QoL was significantly associated with low cough severity.

There are limited published Australian or New Zealand data on health-related resource use in children or adults with bronchiectasis with which to compare our findings. Redding et al. [15] reported that among Indigenous Australian and Alaskan children aged < 8-years, 15% of exacerbations resulted in hospitalization, which is similar to the findings of this study [15]. In the general Australian child population GP attendance was 3.8 visits/year per child between 2012 and 2015 [16], with acute respiratory illness (ARI) the most common reason for presentation. In a cohort of urban Indigenous children aged < 5-years, health service utilisation for ARI was 3.6 visits per child-year [17] with an annual mean cost in 2017 Australian dollars of $991 (95% confidence interval 514–1468) per child [18]. Thus children with bronchiectasis are seeking health care as frequently as young children with common ARIs and, given the predominance of specialist reviews and hospitalizations, the annual cost of bronchiectasis in children is likely to be substantially higher.

Antibiotic use is high in this cohort, although some may be associated with long-term maintenance courses, such as with azithromycin, rather than additional prescriptions for new exacerbations each month. Nevertheless, the prescription rate of 46.0 per 100 child-months (or 552 per 100 child-years) in this cohort is much higher than the antibiotic prescription rate of 41.4 per 100 child-years that was reported for all Australian children aged 0–12 years in 2013 [19]. Of all Australian children dispensed antibiotics for systemic use in 2013, 53% received one systemic antibiotic course, 22% received two, 11% had three and the remainder received four or more. In New Zealand, during 2010–2014, the mean number of antibiotic courses dispensed to children aged < 6-years approximated 190 per 100 child-years, with dispensing rates 29–35% higher among Maori/Pacific Islanders compared to New Zealand European children [20]. The higher frequency of antibiotic use among exacerbations during winter that was identified in this study is consistent with overall trends of antibiotics prescribing to Australian children in primary care settings [21].

In any given month, one-quarter of the children in the cohort missed school or childcare due to their bronchiectasis. In a recent study of Alaskan Native children aged 7–17 years with bronchiectasis, 15/34 missed school in the previous 12-months because of respiratory illnesses [22]. A systematic review of social and economic consequences of childhood asthma [23] reported children with asthma were absent from school for 2.1–14.8 days per year. This compares with an average 12-days per child-year in our study. Chronic school absenteeism is associated with suboptimal school achievement [24], which can lead to long-term social and economic disadvantage. Children with chronic illness often demonstrate worse school outcomes than their peers without chronic illness [25]. Furthermore, parents/carers frequently take time from work to care for their child, resulting in lost productivity and income.

The overall high QoL and low cough scores we observed are not surprising given that they were obtained during routine clinical visits, as found in another Brisbane study reporting similar QoL scores for children in a stable state [7]. Our finding that both PC-QoL and CC-QoL scores were negatively associated with cough severity is consistent with PC-QoL significantly declining during exacerbations [7].

This study has several limitations. As all symptom and resource use data were parent-reported and data were collected on a monthly basis, there is a potential for parent/guardian misclassification and recall bias respectively. Future studies could reduce the risk of recall bias, and improve the accuracy of findings, by using medical records to confirm parent-reported data and/or having more frequent follow-up. With respect to the representativeness of the study sample; the differences in baseline characteristics between those participants who completed all monthly follow-ups, and those that did not, suggest that younger children and children with poor birth outcomes were over-represented among the data obtained. Redding et al. [15] reported that among Indigenous children with bronchiectasis, younger children (aged < 3-years) were more likely to be hospitalized than older children. Poor birth outcomes are recognized risk factors for respiratory disease [26] and it is plausible that poor birth outcomes may be associated with higher resource use as a result of earlier and more severe respiratory disease.

The study was conducted in three centers where quarterly clinical reviews are standard care and where the relationship between clinicians and their patients is such that families have ongoing access to advice and support when needed. Hence our findings may apply only to centers with similar practices. Furthermore, whilst the clinical characteristics of our children are similar to other bronchiectasis studies conducted in the same study centres [9], the study findings may not be generalisable to children with bronchiectasis elsewhere [27, 28] given reported differences in clinical presentations and underlying etiology. Nevertheless, as shown in Table 3, the attendance at scheduled visits following enrolment was only about 50% for reasons that are uncertain. It may reflect visits that were scheduled when the child was well and the parent/carer did not consider attendance necessary and/or the competing demands of families with respect to work and school, a concern reported by New Zealand mothers of children with bronchiectasis [29]. The small number of parents/carers and children who completed QoL and cough severity questionnaires is likely to have biased our findings, particularly as most questionnaires were completed at clinic visits. It is plausible that the QoL of parents/carers and children who regularly attend scheduled visits and receive ongoing specialist care may be better than those who do not [30]. Alternatively, those not attending may have milder disease. Finally, the tool used to measure cough severity has been reported to have some limitations with respect to its correlation with cough frequency. However there are few other validated tools for assessing cough severity in children [31] and this tool has been used repeatedly in other paediatric respiratory studies, enabling comparisons to be made between studies [32–34].

## Conclusions

To our knowledge, this study is the first to provide disease burden estimates by measuring healthcare resource use for managing pediatric bronchiectasis. It suggests resource use is substantial. Further research is needed to more precisely estimate the cost of disease to families, the healthcare sector and third parties in a broader population of children with bronchiectasis. Such data are critical for informing policy and designing and evaluating interventions to reduce the burden of disease.

## Additional file

Additional file 1: **Table S1.** Parent and child-reported cough severity scores at time of clinic visits. **Table S2.** Correlations between parent and child-reported quality-of-life (QoL) and parent and child-reported cough severity at baseline and months 3, 6, 9 and 12. **Table S3.** Comparison of baseline characteristics between those participants who completed all 12-monthly follow-up tasks and those participants who did not. (DOCX 21 kb)

## Data Availability

The datasets used and/or analyzed during the current study are available from the corresponding author on reasonable request.

## Abbreviations

ARI: Acute respiratory illness
CC-QoL: Chronic cough quality of life
ED: Emergency department
GP: General practitioner
HR-QoL: Health-related quality of life
IQR: Interquartile range
NHMRC: National Health & Medical Research Council
NZD: New Zealand Dollar
PC-QoL: Parent-cough-specific quality of life
QoL: Quality of life
SD: Standard deviation
