# Supplementary material for: Health-resource use and quality of life in children with bronchiectasis: a multi-center pilot cohort study
Source: BMC Health Serv Res. 2019 Aug 13;19:561. doi: 10.1186/s12913-019-4414-5 (PMC6693266; doi:10.1186/s12913-019-4414-5)
Supplement: Supplementary file 1 — Table S1. Parent and child-reported cough severity scores at time of clinic visits. Table S2. Correlations between parent and child-reported quality-of-life (QoL) and parent and child-reported cough severity at baseline and months 3, 6, 9 and 12. Table S3. Comparison of baseline characteristics between those participants who completed all 12-monthly follow-up tasks and those participants who did not. (DOCX 21 kb) [file 12913_2019_4414_MOESM1_ESM.docx]

**Additional File 1**

Table S1: Parent and child-reported cough severity scores at time of clinic visits

|  | Baseline | | Month 3 | | Month 6 | | Month 9 | | Month 12 | |
| --- | --- | --- | --- | --- | --- | --- | --- | --- | --- | --- |
|  | Parent  (n=85) | Child  (n=49) | Parent  (n=85) | Child  (n=52) | Parent  (n=85) | Child  (n=54) | Parent  (n=85) | Child  (n=57) | Parent  (n=85) | Child  (n=57) |
| Cough severity scores^¶^ |  |  |  |  |  |  |  |  |  |  |
| 0 *(least severe)* | 24 | 14 | 17 | 5 | 19 | 8 | 17 | 6 | 18 | 9 |
| 1 | 18 | 5 | 10 | 2 | 8 | 6 | 8 | 6 | 6 | 4 |
| 2 | 16 | 11 | 7 | 8 | 14 | 2 | 7 | 1 | 15 | 4 |
| 3 | 7 | 4 | 4 | 2 | 8 | 5 | 6 | 3 | 4 | 4 |
| 4 | 8 | 1 | 5 | 2 | 3 | 2 | 5 | 4 | 6 | 4 |
| 5 *(most severe)* | 1 | 0 | 1 | 1 | 2 | 2 | 0 | 0 | 0 | 0 |
| Missing | 11 | 14 | 41 | 32 | 31 | 29 | 42 | 37 | 36 | 32 |
| Median (IQR) | 1.0  (0.0-2.0) | 1.0  (0.0-2.0) | 1.0  (0.0-2.0) | 2.0  (0.5-2.5) | 1.5  (0.0-2.0) | 1.0  (0.0-3.0) | 1.0  (0.0-3.0) | 1.0  (0.0-3.0) | 2.0  (0.0-2.0) | 1.0  (0.0-3.0) |

^¶^0 = no cough, 1 = cough for one or two short periods only, 2 = cough for more than two short periods, 3 = frequent coughing which does not interfere with school or other normal activities, 4 = frequent coughing which does interfere with school or other normal activities, 5 = cannot perform most usual activities due to severe coughing.

Table S2. Correlations between parent and child-reported quality-of-life (QoL) and parent and child-reported cough severity at baseline and months 3, 6, 9 and 12

| Parent QoL – child QoL | n | Pearson coefficient | p-value |
| --- | --- | --- | --- |
| Baseline | 33 | 0.67 | <0.001 |
| Month 3 | 19 | 0.67 | 0.002 |
| Month 6 | 25 | 0.75 | <0.001 |
| Month 9 | 19 | 0.73 | <0.001 |
| Month 12 | 25 | 0.62 | <0.001 |
| Parent cough severity – child cough severity | n | Spearman coefficient | p-value |
| Baseline | 35 | 0.77 | <0.001 |
| Month 3 | 19 | 0.85 | <0.001 |
| Month 6 | 24 | 0.93 | <0.001 |
| Month 9 | 18 | 0.73 | <0.001 |
| Month 12 | 24 | 0.95 | <0.001 |
| Child QoL – child cough severity | n | Spearman coefficient | p-value |
| Baseline | 35 | -0.59 | <0.001 |
| Month 3 | 20 | -0.63 | 0.003 |
| Month 6 | 25 | -0.78 | <0.001 |
| Month 9 | 19 | -0.79 | <0.001 |
| Month 12 | 24 | -0.76 | <0.001 |
| Parent QoL – parent cough severity | n | Spearman coefficient | p-value |
| Baseline | 70 | -0.69 | <0.001 |
| Month 3 | 40 | -0.65 | <0.001 |
| Month 6 | 54 | -0.75 | <0.001 |
| Month 9 | 42 | -0.57 | <0.001 |
| Month 12 | 45 | -0.83 | <0.001 |

Table S3: Comparison of baseline characteristics between those participants who completed all 12-monthly follow-up tasks and those participants who did not.

| Baseline characteristics, n (%) | Monthly follow-up tasks complete  *n = 67* | Monthly follow-up tasks incomplete  *n = 18* |
| --- | --- | --- |
| Enrolment site | | |
| Brisbane | 38 (56.7) | 9 (50.0) |
| Darwin | 9 (11.9) | 4 (22.2) |
| Auckland | 21 (31.3) | 5 (27.8) |
| Age at enrolment | | |
| <5-years | 15 (22.4) | 1 (5.6) |
| 5-9 years | 25 (37.3) | 13 (72.2) |
| ≥10-years | 26 (38.8) | 4 (22.2) |
| Missing | 1 (1.5) | 0 (0.0) |
| Sex | | |
| Male | 33 (49.3) | 10 (55.6) |
| Female | 34 (50.8) | 8 (44.4) |
| Indigenous status | | |
| Indigenous Australian | 5 (7.5) | 2 (11.1) |
| Indigenous New Zealander | 22 (32.8) | 5 (27.8) |
| Non-Indigenous | 40 (59.7) | 11 (61.1) |
| Gestational age | | |
| <37-weeks | 19 (28.4) | 1 (5.6) |
| ≥37-weeks | 47 (70.2) | 17 (94.4) |
| Birthweight | | |
| <2500 grams | 17 (25.4) | 1 (5.6) |
| ≥2500 grams | 50 (74.6) | 17 (94.4) |
| Number of lobes affected | | |
| 1 | 7 (10.5) | 1 (5.6) |
| 2 | 21 (31.3) | 12 (66.7) |
| 3 | 1 (26.9) | 2 (11.1) |
| 4 | 5 (7.5) | 1 (5.6) |
| 5 | 4 (6.0) | 2 (11.1) |
| 6 | 1 (1.45) | 0 (0.0) |
| Missing | 11 (16.4) | 0 (0.0) |
| Aetiology of bronchiectasis | | |
| Post-infectious | 38 (56.7) | 13 (72.2) |
| Idiopathic | 11 (16.4) | 3 (16.7) |
| Aspiration | 5 (7.5) | 2 (11.1) |
| Primary immunodeficiency | 4 (6.0) | 0 (0.0) |
| Primary ciliary dyskinesia | 3 (4.5) | 0 (0.0) |
| Other | 6 (9.0) | 0 (0.0) |
